# Effects of the Bragg peak degradation due to lung tissue in proton therapy of lung cancer patients

**DOI:** 10.1186/s13014-019-1375-0

**Published:** 2019-10-25

**Authors:** Kilian-Simon Baumann, Veronika Flatten, Uli Weber, Stefan Lautenschläger, Fabian Eberle, Klemens Zink, Rita Engenhart-Cabillic

**Affiliations:** 1 University Medical Center Giessen-Marburg, Department of Radiotherapy and Radiooncology, Marburg, Germany; 2 University of Applied Sciences, Institute of Medical Physics and Radiation Protection, Giessen, Germany; 3 GSI Helmholtzzentrum für Schwerionenforschung, Biophysics Division, Darmstadt, Germany; 4 Marburg Ion-Beam Therapy Center (MIT), Marburg, Germany; 5 Frankfurt Institute of Advanced Studies – FIAS, Frankfurt, Germany

**Keywords:** Proton therapy, Lung modulation, Bragg peak degradation, Treatment planning

## Abstract

**Purpose:**

To quantify the effects of the Bragg peak degradation due to lung tissue on treatment plans of lung cancer patients with spot scanning proton therapy and to give a conservative approximation of these effects.

**Methods and materials:**

Treatment plans of five lung cancer patients (tumors of sizes 2.7–46.4 cm^3^ at different depths in the lung) were optimized without consideration of the Bragg peak degradation. These treatment plans were recalculated with the Monte Carlo code TOPAS in two scenarios: in a first scenario, the treatment plans were calculated without including the Bragg peak degradation to reproduce the dose distribution predicted by the treatment-planning system (TPS). In a second scenario, the treatment plans were calculated while including the Bragg peak degradation. Subsequently, the plans were compared by means of D_mean_, D_98%_ and D_2%_ in the clinical target volume (CTV) and organs at risk (OAR). Furthermore, isodose lines were investigated and a gamma index analysis was performed.

**Results:**

The Bragg peak degradation leads to a lower dose in the CTV and higher doses in OARs distal to the CTV compared to the prediction from the TPS. The reduction of the mean dose in the CTV was − 5% at maximum and − 2% on average. The deeper a tumor was located in the lung and the smaller its volume the bigger was the effect on the CTV. The enhancement of the mean dose in OARs distal to the CTV was negligible for the cases investigated.

**Conclusions:**

Effects of the Bragg peak degradation due to lung tissue were investigated for lung cancer treatment plans in proton therapy. This study confirms that these effects are clinically tolerable to a certain degree in the current clinical context considering the various more critical dose uncertainties due to motion and range uncertainties in proton therapy.

## Background

Since proposed for radiation therapy, ion beams are of increasing interest in radiation oncology [1, 2]. In homogeneous materials the dose profile of heavy charged particles such as protons consists of a low dose plateau at small depths followed by the so-called Bragg peak where most of the dose is deposited. The Bragg peak is followed by a sharp distal fall-off. This finite range and sharp distal fall-off of the dose deposition as well as the comparable low entrance dose lead to a reduction in the integral dose. These advantages of protons in radiation therapy lead to the possibility of a conformal dose distribution in the target while sparing surrounding healthy tissue [3]. Mainly two approaches for the use of proton therapy have evolved [4]: 1) to escalate the dose in the tumor while allowing the same dose to organs at risk (OAR) compared to conventional photon therapy and 2) keeping the target dose constant compared to conventional photon therapy and reducing the dose deposited to OARs as much as possible. Both approaches can be considered for the treatment of lung cancer patients with protons. On the one hand, it has been shown in some studies that a dose escalation in the tumor improves local control and survival in non-small cell lung cancer (NSCLC) patients [4]. On the other hand, the sparing of healthy tissue is of interest for tumors in difficult anatomies where the tumor is located near to sensitive structures or even enclosed by OARs. This is often the case for lung cancer patients due to the possible proximity of OARs like the heart, esophagus, trachea, large blood vessels and the spinal cord [4].

For early-stage NSCLC patients the outcomes achieved with proton therapy are similar to those achieved with stereotactic body radiotherapy (SBRT) [4] despite excellent dose distributions and sparing of OARs. This holds especially for small peripheral lesions, since these are mostly located far from critical structures (except for ribs and chest wall) and hence a sparing of OARs is well achievable with SBRT. However, for larger tumors, especially those located near to OARS, proton therapy might be superior to SBRT.

For locally advanced (stage III) lung cancer patients, virtual clinical studies showed that in proton plans it is possible to reduce the dose deposition in normal tissue, especially in the heart, compared to photon plans [3–5]. Additionally, a phase II study with 44 patients showed an enhanced median survival in a combined radio-chemotherapy when irradiating with protons compared to photons. The patients treated with protons showed minimal grade 3 toxicities [4, 6].

In addition to the debatable benefits of proton therapy compared to SBRT in lung cancers, some difficulties in treating lung cancers with protons arise due to the underlying physics as well as the technical application of the proton beam. One of the main issues is the range of protons that depends on the material in the beam path: in case that the patient’s anatomy changes and hence is different during the treatment compared to the treatment-planning process, the range and hence the dose deposition can be different to that predicted by the TPS. Hence, the outcome of the treatment is vulnerable to changes in the anatomy of the patient as the patient moves, is not optimally positioned or the anatomy of the patient changes between fractions, e.g. due to a shrinkage of the tumor or weight loss [7–10]. Especially the change in the anatomy between fractions causes a need in plan adaption strategies for proton therapy [11].

Another issue is that the range of protons is predicted based on X-ray CT images while the conversion of stopping powers from X-ray CT images is inaccurate [12, 13]. Furthermore, uncertainties in the dose deposited arise from uncertainties in biological effectiveness models [7, 14].

A crucial topic in the proton therapy of lung cancer patients is motion management since interplay effects due to respiratory motion or motion of the heart combined with the precise application of actively scanned proton beams can lead to a severe underdosage of the target volume [15–20].

Another uncertainty in proton therapy of lung cancer patients arises from the characteristics of the lung tissue itself: The heterogeneous structure of the lung tissue leads to a degradation of the Bragg peak and to a wider distal fall-off [21, 22]. If this degradation is not considered correctly during the treatment planning of lung cancer patients, it might lead to an underdosage of the target volume and an overdosage of normal tissue distal to the target volume [22, 23]. Although this degradation has been described in numerous works [24–29], it cannot be considered in the clinical treatment-planning process and dose calculation on treatment-planning CT images. The reason is that due to the restricted resolution of treatment-planning CTs, the microscopic structure of lung tissue is not resolved sufficiently and a more homogeneous tissue distribution is predicted [26, 30].

Baumann et al. [29] introduced and extensively tested an efficient method to consider the Bragg peak degradation on the base of typically used treatment-planning CT data in Monte Carlo codes by applying a density modulation to voxels associated with the lung. Flatten et al. [31] used this model to estimate the effects of the Bragg peak degradation based on a phantom study where spherical tumors of different sizes were placed at different depths in the lung and the underdosage of the target volume was quantified. The result showed that the underdosage of the target volume increases with an increasing depth of the tumor in lung and a decreasing tumor volume. The maximum underdosage in terms of the mean dose was − 15% compared to the dose distribution predicted by the treatment-planning system that did not consider the Bragg peak degradation.

In this study the effects of the Bragg peak degradation were investigated on clinical cases for various anatomical locations of the tumor in the lung and different treatment plans. We chose simple field configurations so that the results can be used by a large variety of proton centers. The goal is to give upwards estimations for the dose uncertainty in the target volume and OARs. For that, we included also extreme cases (e.g small tumor volumes and large depths in lung) to quantify the maximum degradation effect in realistic patient anatomies.

## Methods and materials

### Selection of patients

**Fig. 1:**
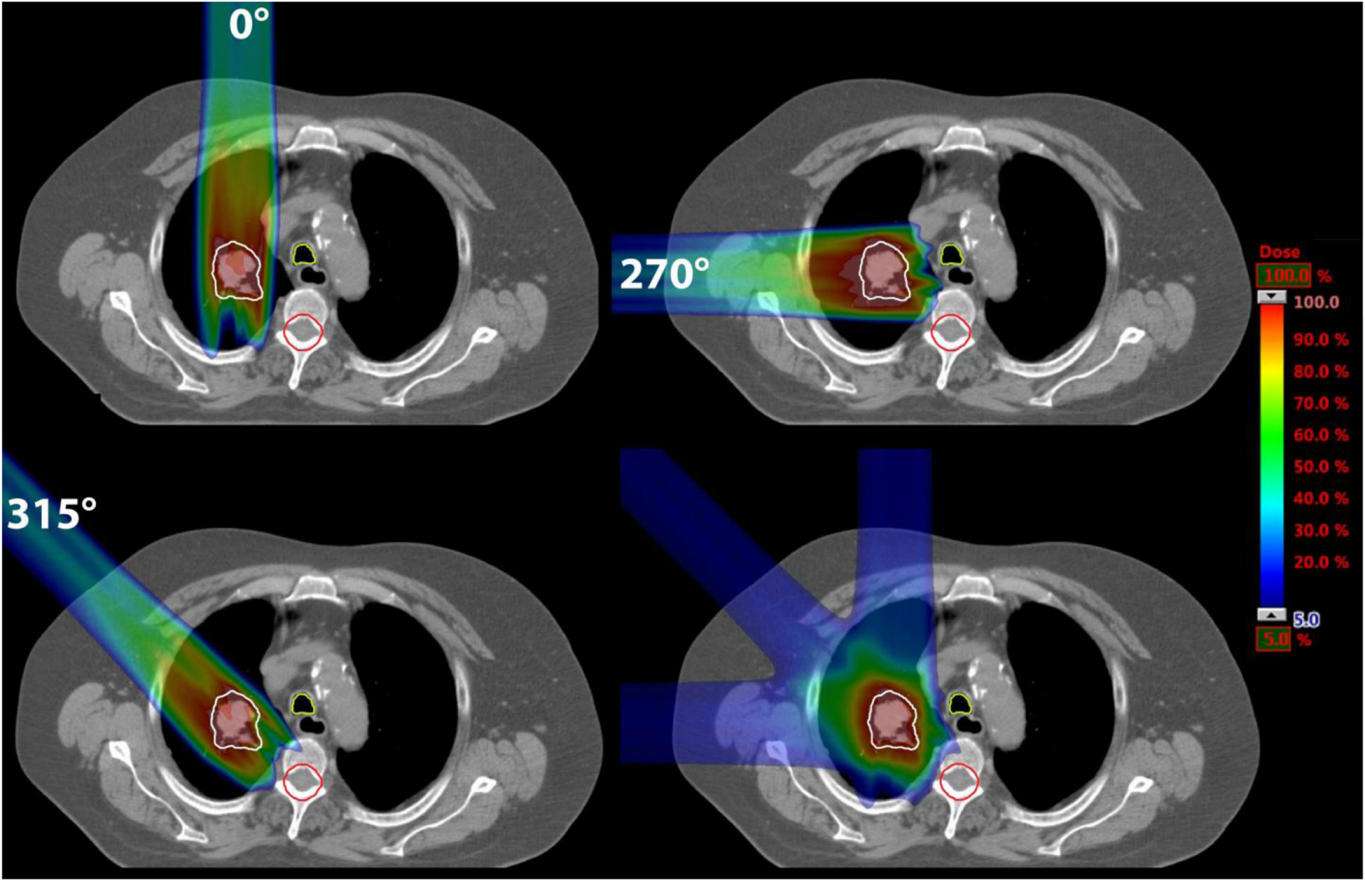
CT slices of one exemplary patient (patient 1): for the beam directions 0°, 270° and 315° plans were optimized individually consisting of one single field. On the bottom right the sum of these plans is shown. The CTV is marked in white, trachea in light green and spinal cord in red. On the right a color bar is given indicating the relative dose

We investigated five exemplary clinical cases with tumor volumes between 2.7 cm^3^ and 46.4 cm^3^. The tumors were located in the right lung in the upper lobe (two cases) or the central lobe (three cases). We chose the clinical cases to have tumors located in the center of the lung as well as tumors that are located near to soft tissue or OARs. In doing so we are able to investigate different depth of the tumor in the lung as well as the effects of the Bragg peak degradation on surrounding normal tissue and OARs. For two cases the tumor was located near to the spinal cord. No tumor was located directly at the thorax wall in order to always have lung tissue between the thorax wall and the tumor and hence in the beam path. The patients were originally treated with photons and retrospectively re-planned with protons for this study. We used different beam directions in the proton plans to generate different path lengths in the lung (see Fig. 1 and Table 1).

**Table 1:** Volumes of the CTVs and lungs for the five patients and minimum and maximum diameter of the CTVs as well as depths in lung of the CTVs for each beam direction. The depth of the CTV corresponds to the path length through lung tissue for the spot at the isocenter

Patient	Lung volume in cm^3^	Volume of CTV in cm^3^	min./max. diameter of CTV in cm	Depth of CTV in cm for different beam directions		
				0°	270°	315°
1	2294	46.4	2.9/4.2	6.2	3.3	3.6
2	1882	4.2	1.8/2.3	1.8	2.1	1.5
3	1705	32.1	3.5/5.2	12.2	9.2	9.2
4	1780	6.2	2.2/2.4	6.9	2.7	5.3
5	1600	2.7	1.6/2.0	4.5	3.8	3.6

As described in the introduction, small tumor volumes as investigated in this study might not benefit from proton therapy compared to SBRT and hence tend to be less relevant for proton therapy. Yet, small tumor volumes have been treated with protons at different centers [32–34], with volumes going down to only 1 cm^3^. Furthermore, Flatten et al. [31] showed that the effects of the Bragg peak degradation increase with a decreasing tumor volume. Thus, we investigated these small tumors as well, in particular to derive an upwards estimation for larger and thus clinically more relevant tumor volumes.

### Treatment planning

All treatment plans were optimized with Eclipse v.13.7 (VARIAN) using the non-linear universal proton optimizer, v.13.7.15. The total prescribed dose was 30 Gy (RBE) and the only planning objective was to deliver at least 95% of the prescription dose to at least 98% of the planning target volume (PTV). For small tumor volumes we accepted hot spots (up to 115% of the prescribed dose) in the PTV. The PTV was the clinical target volume (CTV) plus an isotropic margin of 3 mm, although most PTV concepts proposed in the literature [7, 34–36] are field specific and account for uncertainties of the proton’s range or the positioning of the patient. However, in the majority of clinical situations, an isotropic margin around the CTV is used for treatment planning as done in this study. Additionally, we planned on static CT data, ignoring movements of the anatomy due to respiration in both the planning and dose calculation. By excluding any effects due to motion, positioning of the patient or range uncertainties, we are able to extract the pure effects of the Bragg peak degradation. We analyzed these effects on the CTV and not the PTV, since the CTV is the clinically relevant structure. Since for each optimized plan the dose deposited in the OARs was below any critical value we did not include any constraints for OARs in the treatment-plan optimization.

We decided to investigate simple treatment plans consisting of only one single field coming from either 0°, 270° or 315°. We used different beam directions to generate different depths of the CTV in the lung. The distal spot spacing was 3 mm, the lateral spot spacing was 0.45 times the full-width-half-maximum (FWHM) in air. The FWHM for 70 MeV protons was 32.5 mm and for 221 MeV protons it was 8.1 mm.

**Fig. 2:**
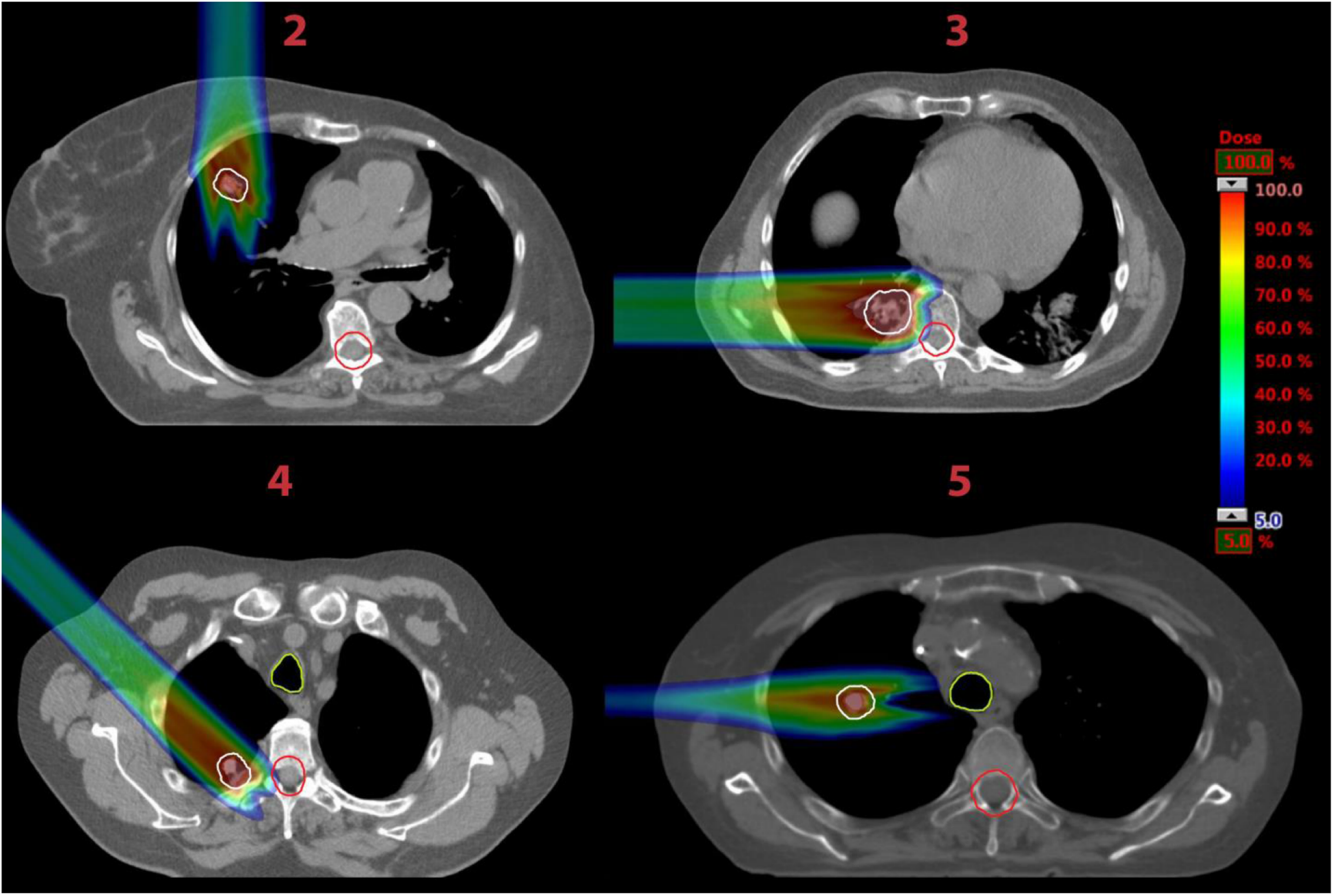
CT slices of the remaining patients. The patient’s numbers are marked in red. For each patient one exemplary plan is shown. The CTV is marked in white, trachea in light green and spinal cord in red. On the right a color bar is given indicating the relative dose

The different beam directions are shown in Fig. 1 for one exemplary patient. As described later in the text, we also made a sum plan of the three single field plans for each patient as shown in Fig. 1 bottom right. In Fig. 2 the remaining four patients are shown with one exemplary treatment plan each.

Although some of these beams may not be the best choice from a clinical point of view (e.g. OARs distal to the PTV, large depth of the tumor in the lung), we decided to investigate these cases anyway to give an upwards estimation for the effects of the Bragg peak degradation also for worst-case scenarios.

The motivation for using simple treatment plans is to highlight the effects of the Bragg peak degradation. Furthermore, there is no gold standard in plan design for lung cancer patients, especially concerning the choice of number of fields and beam directions, although several proton centers have already treated lung cancer patients with protons [6, 32–34]. Thus, in keeping the treatment plans simple, we can assure that the results from this study are usable for as many different proton centers as possible since the dependencies of the Bragg peak degradation (e.g. on the depth of tumor in lung) can be assessed more easily using simple treatment plans compared to complex IMPT plans.

**Fig. 3:**
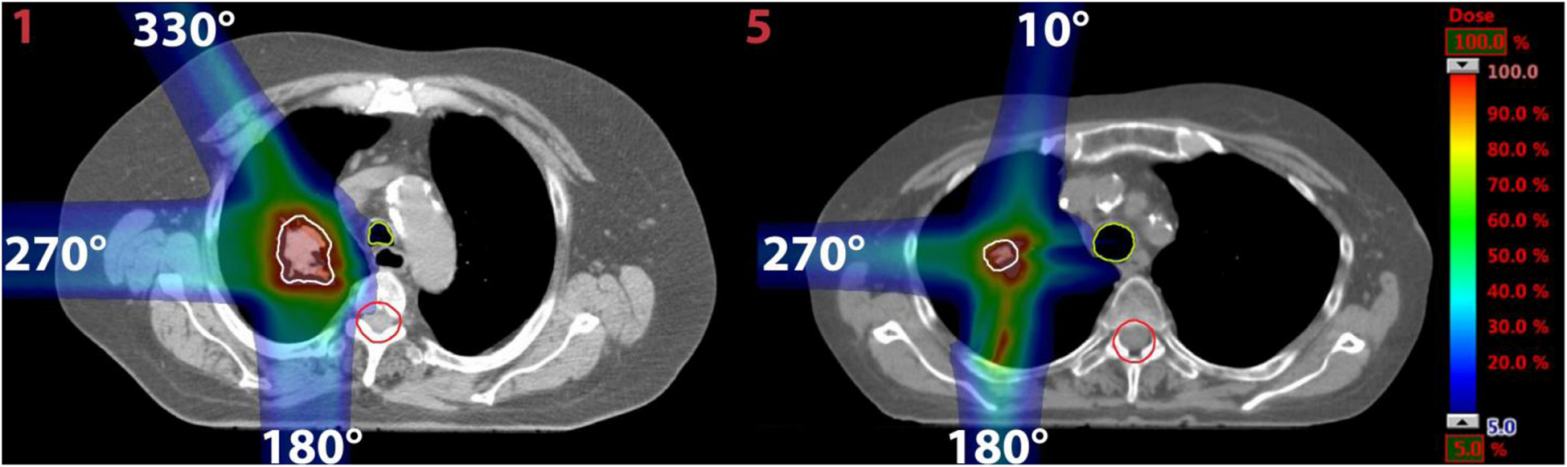
CT slices of the patients 1 and 5 (marked in red numbers) with the dose distributions for the optimized IMPT plans. The CTV is marked in white, trachea in light green and spinal cord in red. On the right a color bar is given indicating the relative dose

In order to assess whether the results from this study can be used to estimate the effects of the Bragg peak degradation for more complex plans, we investigated two IMPT plans, one each for patient 1 and 5 as shown in Fig. 3. The choice fell on these two patients since they have the largest and smallest tumor volume (compare Table 1). Multi-field optimization was enabled to optimize three fields for each plan. The same PTV concept and planning objectives were used as for the simple plans. For patient 1 the beam directions were 180°, 270° and 330°. For patient 5 the beam directions 10°, 180° and 270° were used.

### Simulations

Simulations were performed using the Monte Carlo code TOPAS (Tool for Particle Simulations) version 3.1.p1 [37], a toolkit based on Geant4 (Geometry And Tracking) version geant4–10-03-patch-01 [38]. We used the same beam data in both TOPAS and Eclipse and commissioned these data to match the beam delivery system at the Ion-Beam Therapy Center Marburg. Dose calculation results in water between TOPAS and Eclipse agreed well. The passing rate of the gamma index 1%/1 mm for voxels with dose values greater than 20% of the maximum dose was larger than 98% for single spots. It is known that differences between dose calculation algorithms as used in Eclipse and Monte Carlo codes such as TOPAS exist especially for dose calculations in the lung [39]. Hence, all dose calculations were performed with TOPAS so that differences in the dose calculation between TOPAS and Eclipse do not falsify the results.

**Fig. 4:**
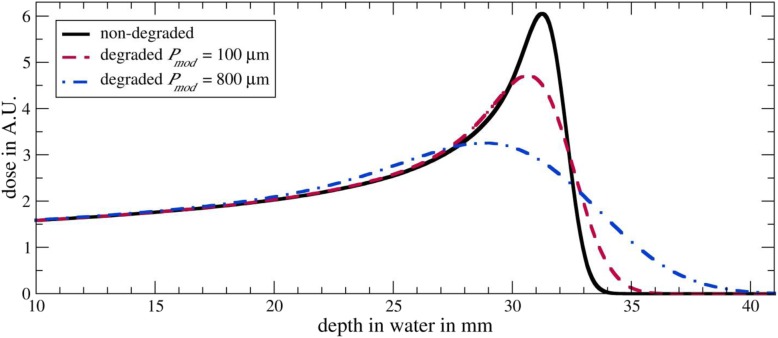
Depth dose curves of protons in water. In black the Bragg curve as predicted by the treatment-planning system that does not consider the Bragg peak degradation due to the lung tissue. In red and blue the degraded Bragg curves based on modulation powers *P*_*mod*_ of 100 μm and 800 μm. The greater the modulation power the broader the Bragg peak and the less steep the distal fall-off

Each treatment plan optimized with Eclipse was recalculated in TOPAS in two scenarios: In the first scenario, each optimized treatment plan was calculated on the original CT data. Hence, this calculation corresponds to the prediction from the treatment-planning system. In a second scenario, the plans were recalculated while considering the Bragg Peak degradation. To do so, we used the mathematical model presented by Baumann et al. [29]. The strength of the Bragg peak degradation is quantified by the material characteristic “modulation power” *P*_*mod*_: The greater the modulation power of a heterogeneous material like lung tissue the broader the Bragg peak and the less steep the distal fall-off as shown in Fig. 4. Based on the modulation power a density distribution can be derived. When modulating the density of each voxel associated with the lung within the patient following this density distribution, the Bragg peak degradation due to the lung tissue is being reproduced [29]. The dose distributions for each plan obtained from using the original CT data (non-modulated case) and when applying the density modulation (modulated case) were compared in means of cumulative dose volume histograms (DVH), mean doses D_mean_, D_98%_ and D_2%_ in the CTV and OARs. Additionally, we investigated the differences in the dose distribution when combining the dose distributions from the single plans (beam directions 0°, 270° and 315°) for each patient to an added-up dose distribution to investigate the influence of the number of irradiated fields on the degradation effects.

In a study by Witt [40] the modulation powers of porcine lungs were measured in proton beams to be in the range from 300 μm to 750 μm. Since the measurements were performed with complete lungs, the measured modulation powers correspond to integrated modulation powers of all the structures of the lung being arranged in the beam that cannot be identified in CT images. The modulation power increases with increasing structure sizes [29]. Thus, the modulation power varies with the position in the lung. In the peripheral region of the lung the modulation power tends to be smaller compared to the central lung where the size of the structures is greater. For almost each measurement the modulation power was in the range from 300 μm to 500 μm with an average of 450 μm. For one measurement the modulation power was 750 μm. However, for this measurement the lung was positioned in a way that a large bronchial structure was in the beam line.

To clarify whether these results obtained with porcine lungs are applicable for human lung tissue, high-resolution CT images with a resolution of 4 μm of human lung tissue samples were investigated by Witt [40] and Baumann et al. [29]. The so-gained modulation powers were in the range from 50 μm to 250 μm. The authors discussed that the preparation of the tissue samples resulted in a noticeable loss of water of up to 37% and hence in a reduction of the sizes of the lung structures. Therefore, the modulation powers gained in this investigation are lower compared to the measurements with porcine lungs.

Both the measurements with porcine lungs and the investigation of human lung tissue samples indicate that the modulation power of lung tissue is in the order of some hundred micrometers. However, until now there is no possibility to determine a patient-specific modulation power for each region of the lung. Therefore, in this study we investigated the effects of the Bragg peak degradation based on modulation powers of 100 μm, 250 μm, 450 μm and 800 μm, covering the whole range of modulation powers found in the measurements of porcine lungs and the investigation of human lung tissue samples with some additional buffer to determine the minimum and maximum degradation effects in exemplary clinical cases. For the IMPT plans we only used a modulation power of 450 μm.

## Results

**Fig. 5:**
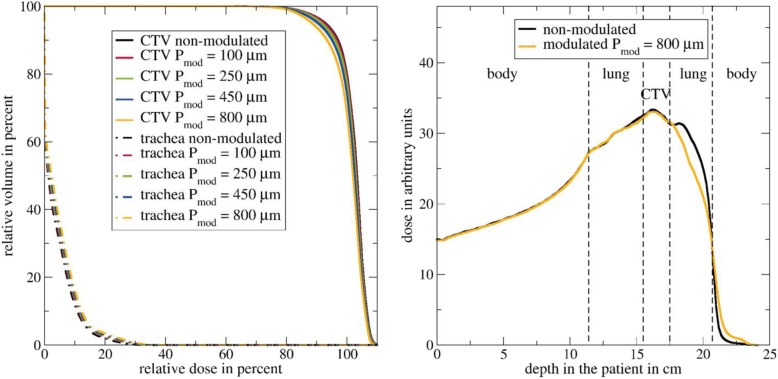
Left side: Cumulative dose-volume histogram for the CTV and trachea for patient 1 and the beam direction 270° for both the non-modulated case and the modulated cases based on modulation powers between 100 μm and 800 μm. Right side: depth dose curve along the center of the beam. The positions of the body, the lung and the CTV are marked by dashed black lines

In Fig. 5 on the left side the DVH for patient 1 and the beam direction 270° is shown for the CTV and the OAR trachea. The Monte Carlo calculated DVH for each volume is shown for the non-modulated case representing the dose distribution predicted by the treatment-planning system and the modulated cases where the Bragg peak degradation based on modulation powers between 100 μm and 800 μm is considered. The dose coverage of the CTV decreases with an increasing modulation power. The dose deposited in the trachea increases with an increasing modulation power. On the right side the depth dose curves along the center of the beam for the non-modulated scenario and the modulated one based on the maximum modulation power of 800 μm are shown. The positions of the body, the lung and the CTV are marked by dashed black lines. We decided to show the results for the extreme modulation power of 800 μm since for a smaller modulation power the effects are not visible as clearly: when entering the body both dose distributions are the same. In the lung the effects of the degradation can be seen resulting in a broader dose curve and a less steep fall-off for the modulated case. Within the CTV there is a slightly higher dose for the non-modulated case. In the lung distal to the CTV there is a significantly higher dose for the non-modulated case. The background is that in this case a spot is used by the TPS where the Bragg peak is located distal to the CTV in order to achieve a sufficient dose coverage within the CTV. This peak is smoothed in the modulated case as described by Flatten et al. [31]. In the body distal to the lung the dose for the modulated case is higher due to the broader fall-off resulting in a larger range and hence a higher dose deposition.

**Fig. 6:**
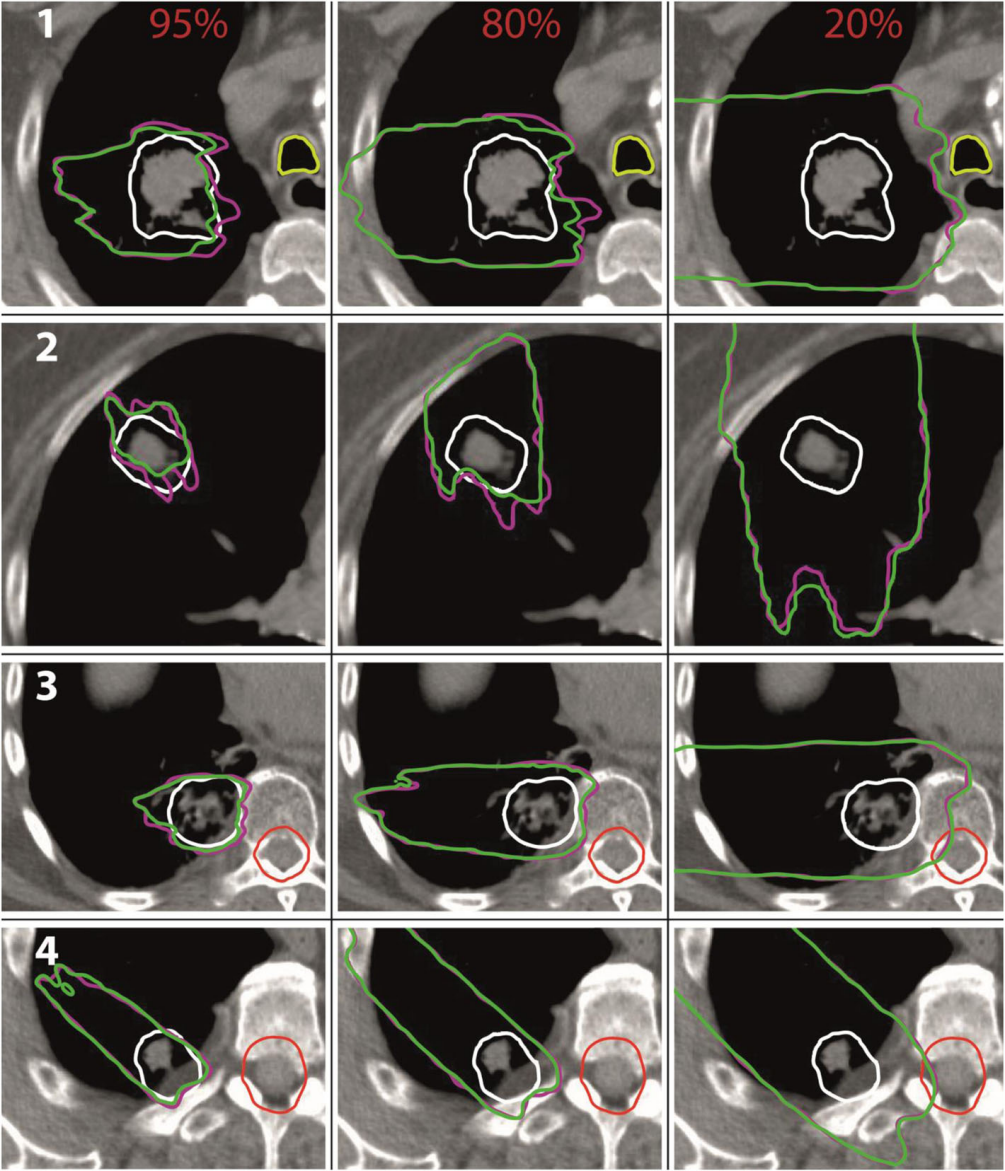
Isodose lines for 95, 80 and 20% of the prescribed dose. In pink for the non-modulated case, in green for the modulated case based on a modulation power of 800 μm. In the first column the 95%, in the middle column the 80% and in the right column the 20% isodose lines. Different patient cases are marked in white numbers. The CTV is marked in white, the trachea in light green and the spinal cord in red

In Fig. 6 exemplary isodose lines [41, 42] for 95, 80 and 20% of the prescribed dose are shown for the non-modulated case (pink) and the modulated case based on a modulation power of 800 μm (green). Additionally, the CTV is marked in white, the trachea in light green and the spinal cord in red. Again, we decided to show the results for the extreme modulation power of 800 μm. The 95% isodose lines are shown in the left column, the 80% in the middle column and the 20% in the right column. In the first line the isodose lines for patient 1 (marked with a white number) are shown for the beam direction 270°. The regions enclosed by the 95 and 80% isodose lines are larger for the non-modulated cases indicating the underdosage of the CTV due to the Bragg peak degradation (compare DVH and depth dose in Fig. 5). The 20% isodose line for the modulated case reaches farther compared to the non-modulated case. The same effects can be seen for all patients.

The greater range of the 20% isodose lines shows the potential risk of an overdosage in OARs distal to the target volume. Especially for patient 3 and 4 in Fig. 6 the isodose lines for 20% of the prescribed dose reach into the spinal cord. However, the additional range of the 20% isodose lines is 2 mm at maximum for these two patients. The maximum shift of each isodose line for the modulated case compared to the non-modulated case for the patients as shown in Fig. 6 are listed in Table 2. A negative sign marks a shorter range compared to the non-modulated case. The range uncertainties of the isodose lines in lung (patient 1 and patient 2 except for the 20% isodose line) are larger compared to those in tissue (patient 3 and 4 all isodose lines and 20% isodose line of patient 1).

**Table 2:** Maximum shift in mm of the isodose lines for the modulated case based on a modulation power of 800 μm compared to the non-modulated case for the patients and beam directions as shown in Fig. 6. A negative sign stands for a shorter range

Patient/beam direction	Maximum range uncertainty in mm for isodose lines		
	95%	80%	20%
1 / 270°	−8	−5	3
2 / 0°	−8	−10	5
3 / 270°	−4	−3	2
4 / 315°	−2	−2	2

To quantify the effects shown in Figs. 5 and 6 in terms of dose, the absolute dose values for the non-modulated case and the differences in percent of the mean dose D_mean_, D_98%_ (the dose that is received by 98% percent of the volume) and D_2%_ between the modulated cases and the non-modulated case for patient 1 are reported in Table 3 for the CTV, the trachea and the spinal cord. The D_98%_ is taken to quantify the minimum dose received by a volume while the D_2%_ is taken to quantify the maximum dose received by a volume. For the OARs the mean dose D_mean_ and the D_2%_ as a quantification of the maximum dose are shown.

**Table 3:** Absolute dose values for the non-modulated case and differences in percent of the mean dose D_mean_, D_98%_ (only for the CTV) and D_2%_ for the CTV and OARs between the modulated and the non-modulated cases for patient 1

Modulation	CTV			Trachea		Spinal cord	
	D_mean_	D_98%_	D_2%_	D_mean_	D_2%_	D_mean_	D_2%_
beam direction: 0° (depth in lung: 6.2 cm)							
D_non-mod_ in Gy (RBE)	29.9	25.7	32.2	0.1	0.5	< 0.1	0.1
100 μm	−0.3%	−0.4%	−0.4%	−1%	+ 1%	0%	− 1%
200 μm	−0.7%	− 1.2%	− 0.8%	0%	+ 1%	0%	− 1%
450 μm	− 1.3%	− 3.0%	− 1.2%	0%	+ 2%	0%	− 1%
800 μm	−2.1%	−4.9%	− 1.8%	0%	+ 2%	+ 1%	− 1%
beam direction: 270° (depth in lung: 3.3 cm)							
D_non-mod_ in Gy (RBE)	30.1	27.3	30.4	1.1	5.6	0.1	0.7
100 μm	−0.2%	−0.1%	−0.2%	+ 7%	+ 9%	+ 2%	+ 6%
200 μm	−0.5%	−1.0%	− 0.3%	+ 12%	+ 13%	+ 2%	+ 9%
450 μm	− 0.5%	−2.2%	− 0.5%	+ 16%	+ 17%	+ 7%	+ 14%
800 μm	− 0.9%	−4.1%	−0.6%	+ 24%	+ 24%	+ 9%	+ 21%
beam direction: 315° (depth in lung: 3.6 cm)							
D_non-mod_ in Gy (RBE)	29.8	26.6	41.6	0.2	1.0	0.1	0.7
100 μm	0.0%	0.0%	+ 0.1%	+ 3%	+ 2%	+ 6%	+ 7%
200 μm	−0.4%	−1.5%	−0.2%	+ 2%	+ 1%	+ 11%	+ 11%
450 μm	−0.8%	−1.5%	− 0.4%	+ 2%	+ 2%	+ 15%	+ 14%
800 μm	− 1.5%	−3.0%	− 0.6%	+ 5%	+ 5%	+ 24%	+ 21%

The mean dose D_mean_, D_98%_ and D_2%_ in the CTV are smaller for the modulated cases compared to the non-modulated case and hence the prediction from the treatment-planning system. The differences increase with an increasing modulation power. The largest differences can be seen for the beam direction 0° (corresponding to the largest depth in lung) and the maximum modulation power of 800 μm. Concerning the OARs trachea and spinal cord, the doses deposited in the modulated cases are greater compared to the non-modulated case for the beam directions 270° and 315°. The differences increase with an increasing modulation power. The maximum difference in the mean dose D_mean_ is + 24% for the trachea as well as the spinal cord. However, these relative deviations correspond to low absolute deviations of 0.3 Gy and < 0.1 Gy, respectively. The largest difference for the D_2%_ value for the trachea is + 24% and + 21% for the spinal cord. These deviations correspond to 1.5 Gy and 0.2 Gy, respectively. It can be seen, that the effects of the Bragg peak degradation on the OARs are almost non-existent for a beam direction 0° since in this case no OAR is positioned distal to the PTV. The effects on the trachea are largest for the beam direction 270°. For the spinal cord the effects are largest for the beam direction 315°.

In Table 4 the absolute dose values and the differences in percent of the mean dose D_mean_, D_98%_ and D_2%_ for the CTV between the modulated cases and the non-modulated case are shown for the patients 2 to 5. The results are given for a modulation power of 800 μm to give an estimation of the maximum effects. Additionally, the results for a more realistic modulation power of 450 μm are given. The deviations between the modulated cases and the non-modulated case for the OARs are as small and negligible as for patient 1 and hence not shown for the other patients. As it is the case with patient 1, the dose coverage of the CTV in the modulated cases is lower compared to the non-modulated case.

**Table 4:** Absolute dose values for the non-modulated case and differences in percent of the mean dose D_mean_, D_98%_ and D_2%_ between the modulated and the non-modulated cases for the CTV and the patients 2 to 5. The modulation powers used in these cases are 450 μm and 800 μm

Patient	Modulation	Beam direction: 0°			Beam direction: 270°			Beam direction: 315°		
		D_mean_	D_98%_	D_2%_	D_mean_	D_98%_	D_2%_	D_mean_	D_98%_	D_2%_
2	D_non-mod_ in Gy (RBE)	29.9	23.4	32.0	30.1	25.2	31.1	30.0	22.1	32.3
	450 μm	−1.8%	−0.7%	−0.2%	− 1.1%	− 0.8%	− 0.7%	−1.1%	− 1.5%	− 0.7%
	800 μm	− 3.1%	− 2.9%	− 3.8%	− 1.9%	−1.4%	− 1.1%	− 2.0%	− 2.5%	−1.2%
3	D_non-mod_ in Gy (RBE)	30.0	25.8	32.2	30.1	28.3	31.5	30.0	27.0	31.9
	450 μm	−1.1%	−1.4%	−0.9%	−0.6%	−2.9%	− 0.5%	− 0.8%	− 2.2%	− 0.8%
	800 μm	− 1.8%	−2.8%	−1.5%	− 1.1%	−5.1%	−0.8%	−1.4%	− 4.2%	− 1.1%
4	D_non-mod_ in Gy (RBE)	30.0	25.7	31.0	30.0	24.2	32.2	30.0	26.8	30.8
	450 μm	−1.3%	−2.5%	−0.8%	−0.6%	−1.2%	− 0.3%	− 0.6%	−1.1%	− 0.4%
	800 μm	− 2.2%	−4.2%	− 1.2%	−1.0%	−2.0%	− 0.5%	−1.0%	−2.0%	− 0.7%
5	D_non-mod_ in Gy (RBE)	30.1	27.2	32.8	30.0	23.7	31.6	30.1	27.9	33.8
	450 μm	−2.6%	−0.5%	−1.1%	−2.0%	−3.0%	− 2.1%	−3.0%	− 1.5%	−3.2%
	800 μm	−4.7%	− 2.8%	−2.1%	− 3.1%	− 4.6%	− 3.2%	− 4.9%	−2.5%	−4.3%

For a modulation power of 800 μm the maximum differences in the mean dose D_mean_ as well as the D_98%_ are roughly − 5%. For the D_2%_ it is about − 4%. The average difference in the mean dose D_mean_ is in the order of − 2%, for D_98%_ it is − 3% and for D_2%_ it is about − 2%.

For a more realistic modulation power of 450 μm the maximum differences in the mean dose D_mean_, the D_98%_ as well as the D_2%_ are roughly − 3%. The average difference in the mean dose D_mean_ is in the order of − 1%, for D_98%_ it is roughly − 2% and for D_2%_ it is − 1%.

Additionally, we looked at the differences in the mean dose D_mean_, D_98%_ and D_2%_ for the CTV between the modulated cases and the non-modulated case when all three plans from the beam directions 0°, 270° and 315° are combined. As for the irradiation with one single field, in the combined scenario with three fields, the differences between the modulated cases and the non-modulated case are at maximum about − 5% for a modulation power of 800 μm. For a modulation power of 450 μm the maximum difference is about − 3%.

In Table 5 the passing rates for the gamma index 3%/1 mm (local) are shown for each patient and the dose distributions based on modulation powers of 450 μm and 800 μm. All voxels with at least 20% of the maximum dose were included in the analysis. We chose to set a small distance-to-agreement since the effect of the Bragg peak degradation leads to a broadening of the Bragg peak and hence a small shift in the dose (compare Figs. 5 and 6). The allowed dose difference was set to 3% since this is roughly the average effect on the mean dose in the CTV for a modulation power of 800 μm. For a modulation power of 800 μm, the minimal passing rate is 90.4% for patient 5 and the beam direction 315° corresponding to the maximum difference in the mean dose (compare Table 3). The average passing rate is 96.8%. For a modulation power of 450 μm the minimum passing rate is 93.1% and the average passing rate is 98.5%. We also investigated the gamma index when including only those voxels with at least 80% of the maximum dose. For this gamma index the minimum passing rate is 84.0% with an average passing rate of 94.6% for a modulation power of 800 μm.

**Table 5:** Passing rates in percent of the gamma index 3%/1 mm including all voxels with at least 20% of the maximum dose for all patients depending on the modulation power and beam direction

Patient	Modulation power	Beam direction		
		0°	270°	315°
1	450 μm	98.5	97.2	99.0
	800 μm	96.4	95.4	97.3
2	450 μm	97.3	99.3	99.9
	800 μm	94.7	95.5	99.2
3	450 μm	99.5	99.8	99.6
	800 μm	98.8	99.0	99.0
4	450 μm	99.9	100	99.9
	800 μm	99.6	100	99.9
5	450 μm	96.0	98.2	93.1
	800 μm	91.7	95.6	90.4

In order to assess whether the results from this study being derived using simple treatment plans can be used to estimate the dose uncertainty due to the Bragg peak degradation on more complex plans like IMPT plans, we investigated two IMPT plans - one each for patient 1 and 5. The reduction of the mean dose D_mean_ of the CTV was − 1% for patient 1 and a modulation power of 450 μm. For patient 5 it was about − 3%. For patient 1, the reduction of the mean dose of the CTV approximately corresponds to the average dose reduction for the simple treatments plans with beam directions 0°, 270° and 315° (compare Table 3). For patient 5 the dose reduction for the IMPT plan is in the order of the maximum effect for the simple treatment plans.

## Discussion

The influence of the Bragg peak degradation due to lung tissue on treatment plans of lung cancer patients was investigated. For all cases the treatment-planning system overestimated the dose delivered to the CTV and in some cases underestimated the dose delivered to distal OARs. This effect increases with an increasing modulation power. The maximum underestimation of the mean dose D_mean_ is − 5% for the CTV and an extreme modulation power of 800 μm. The average underestimation is in the order − 2%. This extreme modulation power of 800 μm can occur in cases where a larger bronchial structure in the lung is positioned in the proton beam. However, for a more realistic modulation power of 450 μm, the underestimation of the mean dose D_mean_ is only about − 3% at maximum. The average underestimation is roughly − 1%.

Concerning the effects on OARs, it was shown that the effects are dependent on the beam direction which defines the relative position between the target volume and OAR for a given anatomy: As shown in Fig. 5 on the right side, the Bragg peak degradation results in a higher dose distal to the Bragg peak. Hence, only OARs distal to the PTV can receive a higher dose than predicted by the treatment-planning system. Due to range uncertainties in proton therapy it would typically be avoided to arrange fields in a way that an OAR is located directly distal to the PTV. Nevertheless, in some cases this is inevitable for example when the patient has been previously irradiated in this region or due to technical limitations of the beam delivery system. Additionally, anatomical characteristics could enforce an irradiation where an OAR is positioned distal to the PTV as it is the case with patient 3 as shown in Fig. 2: for the beam directions 315° and 270° the spinal cord is positioned distal to the PTV. However, for the beam direction 0° the beam crosses the heart and the distance in lung is quite large. Since a patient’s anatomy can oblige to use beams where an OAR is positioned distal to the PTV, we also investigated these cases. The underestimation of the mean dose D_mean_ in the OARs trachea and spinal cord was 0.3 Gy at maximum. For the D_2%_ quantifying the maximum dose deposited in these OARs it was 1.5 Gy at maximum. The resulting enhanced dose deposited to OARs is far from any dose constraints used in the conventional treatment planning. Thus, the effects of the Bragg Peak degradation on OARs distal to the PTV are negligible for the cases investigated. However, in cases where the OAR is located directly distal to the PTV the effects might be larger and significant.

We were able to reproduce the findings from Flatten et al. [31] that the effects of the Bragg peak degradation increase with an increasing depth of the tumor in the lung and a decreasing tumor volume: for example, for patient 1 the underdosage of the CTV increases from − 0.5% to − 1% (for a modulation power of 450 μm) between the beam direction 270° where the tumor depth is 3.3 cm and the beam direction 0° where the depth is 4.6 cm.

When comparing the results from patient 1 for the beam direction 315° with the results from patient 5 for the beam direction 315° one can see that in both cases the tumor is at a depth of 3.6 cm (see Table 1). However, the CTV of patient 1 is with 46.4 cm^3^ much larger compared to patient 5 with a volume of 2.7 cm^3^. The effect of the Bragg peak degradation on the mean dose in the CTV for patient 1 is with − 1% much smaller compared to patient 5 with − 3% (for a modulation power of 450 μm).

Regarding the number of fields used to irradiate the CTV, it was shown that as expected, the effect of the Bragg peak degradation is independent on the number of fields as long as these fields are optimized individually.

Concerning the complexity of the irradiation plans, we decided to investigate simple plans with only one single field as described in the Methods & materials section. By investigating different beam directions, a large variety of scenarios (depth of tumor in lung, OAR distal to the PTV) has been covered and even for the worst cases the underdosage of the CTV was − 5% at maximum for an extreme modulation power of 800 μm and only about − 3% for a more realistic modulation power of 450 μm. To assess whether these results can be applied to more complex treatment plans, we investigated two IMPT plans for a realistic modulation power of 450 μm. For both patients the reduction of the mean dose of the CTV was in the same order compared to the simple treatment plans. This supports the statement that the results found in this study – although being derived using simple treatment plans – can be used to estimate the dose uncertainties due to the Bragg peak degradation for more complex plans.

The passing rate of the gamma index was on average 96.8% for a modulation power of 800 μm and 98.5% for a modulation power of 450 μm. The minimum passing rate for a realistic modulation power of 450 μm was 93.1%. The high passing rate of the gamma index is reasonable because as shown in Figs. 5 and 6 the Bragg peak degradation leads to a shift of the isodose lines. This shift is on average very small and hence covered by the distance-to-agreement in the gamma index. For all cases investigated in this study the passing rate was clinically acceptable. In addition to the finding that the reduction of the mean dose is on average only in the order of − 1% and at maximum − 3% for a realistic modulation power of 450 μm, this supports that the effects of the Bragg peak degradation are clinically tolerable.

What is more, it is well-known in the literature that the relative biological effectiveness (RBE) of protons is larger than 1.1 at the distal part of the Bragg peak [43]. At the moment, this change in RBE is not considered in commonly used treatment-planning systems, hence, this larger biological effect might partially balance out the physical underdosage of the target volume due to the Bragg peak degradation which mainly occurs at the distal end of the Bragg peak and hence the target volume (compare Fig. 5). However, this change in RBE could also potentially increase the effects of the larger dose deposited in normal tissue distal to the Bragg peak.

The Bragg peak degradation due to lung tissue is only one of various issues in proton therapy in general and in proton therapy of lung cancer patients in particular as mentioned in the introduction. Thus, the dose and range uncertainties due to this degradation shall be compared to these other uncertainties in order to quantify its importance in the current clinical context. In a study by Paganetti [7] an overview of range uncertainties is given. Range uncertainties in proton therapy arise – among other things – from measurement uncertainties in water for commissioning (±0.3 mm), patient setup (±0.7 mm) or differences in the dose calculation between the treatment-planning system and Monte Carlo codes as a gold standard for dose calculation (±2 mm). Other reasons for range uncertainties are due to the conversion of x-ray HU to stopping powers (±1% of the range) or biological effects (~ 0.8% of the range or ~ 3 mm [14]). These range uncertainties refer to the range of the 80% distal dose and correspond to average values. Furthermore, these uncertainties might be bigger in lung treatments [7]. The maximum range uncertainties for the 80% isodose lines due to the Bragg peak degradation based on an extreme modulation power of 800 μm found in this study (see Table 2) are 10 mm in lung and 4 mm in tissue and hence are in the order of the mentioned average range uncertainties. Note that the values given by Paganetti [7] are average values while the range uncertainties investigated in this study are maximum values.

Additionally, range and dose uncertainties arise when changes of the anatomy due to weight loss or a shrinkage of the tumor are not accounted for. Szeto et al. [10] analyzed robust intensity modulated treatment plans of 16 patients with locally advanced NSCLC. The treatment dose was recalculated based on daily anatomy variations. Eight patients had an undercoverage of the target volume larger than 2 GyE with a maximum of 12 GyE in terms of the D_99_ (dose that is received by 99% of the target volume). With a prescribed dose of 66 GyE this corresponds to relative deviations in the D_99%_ of 3% to 18%. The maximum difference in the D_98%_ found in this study was 3% for a realistic modulation power of 450 μm.

Another crucial issue in proton therapy of lung cancer patients is motion. Dowdell et al. [20] investigated treatment plans for 5 lung cancer patients. Due to the interplay effects caused by the patient’s motion, the mean dose in the target volume was only 88% to 92% of the prescribed dose. These interplay effects are however highly patient specific.

**Fig. 7:**
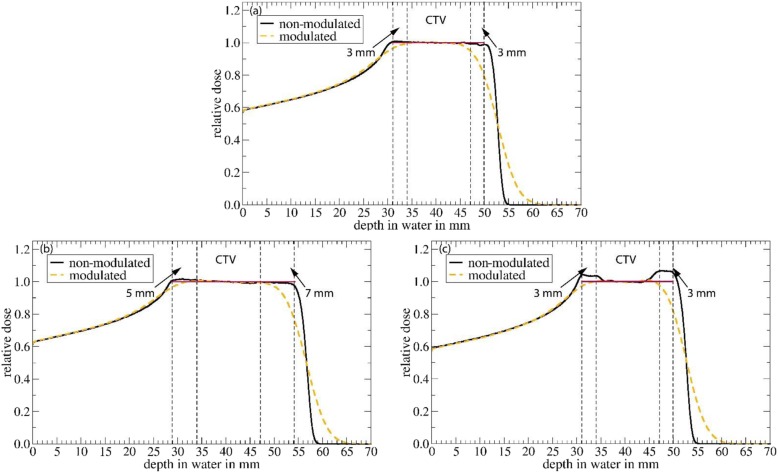
The effects of different PTV concepts. In **a** the effects for a PTV as used in this study consisting of an isotropic margin of 3 mm around the CTV are shown. In **b** for a PTV concept that has a 5 mm margin at the proximal end and a 7 mm margin at the distal end of the CTV. In **c** for the same PTV concept as used in **a**, however, in this case the prescribed dose within this margin is larger than the prescribed dose in the CTV. In black the dose distribution for the non-modulated case and in yellow the dose distribution for the modulated case based on a modulation power of 450 μm. The CTV and the margin as well as the resulting PTV are marked in dashed lines. The red line marks the prescribed dose within the PTV

At last, we want to introduce and discuss two possible PTV concepts to account for and to avoid an underdosage of the target volume due to the Bragg peak degradation: following the range shifts as shown in Fig. 6, one possible PTV concept could be to increase the margin around the CTV at both the distal and proximal end. The effects of such a PTV concept are depicted in Fig. 7: the dose distributions in a water phantom downstream from 80 mm of lung tissue with a modulation power of 450 μm are shown for the non-modulated (black) and the modulated case (yellow). The CTV marked with dashed lines is at depths between 34 mm and 47 mm. In (a) the dose distributions can be seen for a PTV concept as used in this study with an isotropic margin of 3 mm around the CTV. The red line marks the prescribed dose within the CTV and PTV. In (b) the dose distributions are shown for the case where the PTV is the CTV plus a margin of 5 mm at the proximal end and a margin of 7 mm at the distal end. The dose coverage within the CTV and PTV is better compared to (a). The disadvantage of such a PTV concept is that the dose distribution reaches farther and hence leads to a higher integral dose in the normal tissue and maybe OARs distal to the PTV.

To avoid this larger range and additional dose deposited in normal tissue, another PTV concept might be used as depicted in Fig. 7c: in this case the same PTV concept as in (a) is used (isotropic margin of 3 mm around the CTV), however, during the treatment-planning process a larger dose is prescribed within the margin at both the proximal as well as the distal end. In this case we used a 3% larger dose in the proximal and a 6% larger dose in the distal margin. By doing so a comparable dose distribution in the CTV as in Fig. 7b can be achieved, however, the dose deposited in normal tissue is smaller due to the shorter range. Note that such a PTV concept is connected to challenges since it is hard to guarantee a dose homogeneity in such a small volume. Furthermore, a difference of only 3% in dose in such a small volume is in the order of the uncertainties in proton therapy as discussed above, hence, it would hardly be possible to measure this difference in dose (e.g. as part of quality assurance).

For both PTV concepts an exact knowledge of the anatomy (depth of tumor in lung, tumor volume, location of tumor relative to soft tissue) is important to choose the appropriate values for the additional margin (case b) or the additional dose (case c). Furthermore, the knowledge of the modulation power within the lung tissue is important, since this defines the range and dose uncertainties connected to the Bragg peak degradation. Unfortunately, there is currently no solution to determine the modulation power in patients in-vivo. This is a critical issue still to be solved.

Altogether, the effects of the Bragg peak degradation are at maximum about 5% concerning the underestimation of the mean dose D_mean_ in the CTV when optimizing the treatment plan without considering the degradation due to the lung tissue. Compared to the range and dose uncertainties in proton therapy of lung cancer patients due to the addressed reasons, the effects of the Bragg peak degradation are clinically tolerable to a certain degree in the current clinical context. However, these mentioned dose uncertainties are constantly being reduced which might change this clinical context. Hence, a consideration of the Bragg peak degradation could become more relevant in the future and would bring proton therapy for lung cancer patients closer to a high-precision therapy. The effects of the degradation might be accounted for in the treatment-planning process by applying a corresponding PTV concept as suggested in this study. What is more, this PTV concept and hence the dose deposition in the patient could be optimized when having a detailed knowledge of the lung tissue’s modulation power. In our opinion, the exact determination of this modulation power is one crucial issue still to be solved.

## Conclusion

The effects of the Bragg peak degradation due to lung tissue on lung cancer patients were investigated. The maximum effect on the mean dose D_mean_ in the CTV according to this study was about 5% at maximum for an extreme modulation power of 800 μm, a long distance travelled through lung and a small tumor volume. For a more realistic modulation power of 450 μm the maximum effect was only about 3% in terms of D_mean_. For OARs the effect was negligible for the cases investigated. This study confirms that the effects of the Bragg peak degradation are clinically tolerable to a certain degree in the current clinical context considering the various more critical dose uncertainties due to motion and range uncertainties in proton therapy. Furthermore, these effects might be accounted for by using corresponding PTV concepts as suggested in this study.

## Data Availability

The datasets generated during and/or analyzed during the current study are available from the corresponding author on reasonable request.

## Abbreviations

CTV: Clinical target volume
D_2%_: Dose that is received by 2% of a structure’s volume
D_98%_: Dose that is received by 98% of a structure’s volume
D_mean_: Average dose within a structure
IMPT: Intensity modulated proton therapy
OAR: Organ at risk
PTV: Planning target volume
RBE: Relative biological effectiveness
SBRT: Stereotactic body radiation therapy
