# Correction to: Effects of the Bragg peak degradation due to lung tissue in proton therapy of lung cancer patients

**DOI:** 10.1186/s13014-020-1475-x

**Published:** 2020-02-03

**Authors:** Kilian-Simon Baumann, Veronika Flatten, Uli Weber, Stefan Lautenschläger, Fabian Eberle, Klemens Zink, Rita Engenhart-Cabillic

**Affiliations:** 1 University Medical Center Giessen-Marburg, Department of Radiotherapy and Radiooncology, Marburg, Germany; 2 University of Applied Sciences, Institute of Medical Physics and Radiation Protection, Giessen, Germany; 3 GSI Helmholtzzentrum für Schwerionenforschung, Biophysics Division, Darmstadt, Germany; 4 Marburg Ion-Beam Therapy Center (MIT), Marburg, Germany; 5 Frankfurt Institute of Advanced Studies – FIAS, Frankfurt, Germany

**Correction to: Radiat Oncol**

**https://doi.org/10.1186/s13014-019-1375-0**

Following publication of the original article [1], we have been notified that the below text parts of the Discussion chapter should be changed. Currently the text is as follows:

**Discussion**

The influence of the Bragg peak degradation due to lung tissue on treatment plans of lung cancer patients was investigated. For all cases the treatment-planning system overestimated the dose delivered to the CTV and in some cases underestimated the dose delivered to distal OARs. This effect increases with an increasing modulation power. The maximum underestimation of the mean dose Dmean is − 5% for the CTV and an extreme modulation power of 800 μm. The average underestimation is in the order − 2%. This extreme modulation power of 800 μm can occur in cases where a larger bronchial structure in the lung is positioned in the proton beam. However, for a more realistic modulation power of 450 μm, the underestimation of the mean dose Dmean is only about − 3% at maximum. The average underestimation is roughly − 1%.

Altogether, the effects of the Bragg peak degradation are at maximum about 5% concerning the underestimation of the mean dose Dmean in the CTV when optimizing the treatment plan without considering the degradation due to the lung tissue.

The above-mentioned text should be corrected as per below:

**Discussion**

The influence of the Bragg peak degradation due to lung tissue on treatment plans of lung cancer patients was investigated. For all cases the treatment-planning system overestimated the dose delivered to the CTV and in some cases underestimated the dose delivered to distal OARs. This effect increases with an increasing modulation power. The maximum overestimation of the mean dose Dmean is 5% for the CTV and an extreme modulation power of 800 μm. The average overestimation is in the order 2%. This extreme modulation power of 800 μm can occur in cases where a larger bronchial structure in the lung is positioned in the proton beam. However, for a more realistic modulation power of 450 μm, the overestimation of the mean dose Dmean is only about 3% at maximum. The average overestimation is roughly 1%.

Altogether, the effects of the Bragg peak degradation are at maximum about 5% concerning the overestimation of the mean dose Dmean in the CTV when optimizing the treatment plan without considering the degradation due to the lung tissue.
